# Social Media and Social Functioning in Psychosis: A Systematic Review

**DOI:** 10.2196/13957

**Published:** 2019-06-28

**Authors:** Jone Bjornestad, Wenche Ten Velden Hegelstad, Henrik Berg, Larry Davidson, Inge Joa, Jan Olav Johannessen, Ingrid Melle, Helen J Stain, Ståle Pallesen

**Affiliations:** 1 Department of Social Studies Faculty of Social Sciences University of Stavanger Stavanger Norway; 2 Network for Clinical Research in Psychosis Stavanger University Hospital Stavanger Norway; 3 Norsk Lærer Akademi University College Bergen Norway; 4 School of Medicine Yale University New Haven, CT United States; 5 Institution for Social and Policy Studies Yale University New Haven, CT United States; 6 Network for Medical Sciences Faculty of Health University of Stavanger Stavanger Norway; 7 Norwegian Centre for Mental Disorders Research Faculty of Medicine University of Oslo Oslo Norway; 8 School of Social and Health Sciences Leeds Trinity University Leeds United Kingdom; 9 Department of Psychosocial Science University of Bergen Bergen Norway

**Keywords:** psychosis, schizophrenia, social media, social functioning, measures, assessment, systematic review

## Abstract

**Background:**

Individuals with psychosis are heavy consumers of social media. It is unknown to what degree measures of social functioning include measures of online social activity.

**Objective:**

To examine the inclusion of social media activity in measures of social functioning in psychosis and ultrahigh risk (UHR) for psychosis.

**Methods:**

Two independent authors conducted a search using the following electronic databases: Epistemonikos, Cochrane Database of Systematic Reviews, Database of Abstracts of Reviews of Effects, MEDLINE, Embase, and PsycINFO. The included articles were required to meet all of the following criteria: (1) an empirical study published in the English language in a peer-reviewed journal; (2) the study included a measure of objective or subjective offline (ie, non-Web-mediated contact) and/or online social functioning (ie, Web-mediated contact); (3) the social functioning measure had to be used in samples meeting criteria (ie, Diagnostic and Statistical Manual of Mental Disorders or International Classification of Diseases) for a psychotic disorder or UHR for psychosis; and (4) the study was published between January 2004 and February 2019. Facebook was launched as the first large-scale social media platform in 2004 and, therefore, it is highly improbable that studies conducted prior to 2004 would have included measures of social media activity.

**Results:**

The electronic search resulted in 11,844 distinct articles. Full-text evaluation was conducted on 719 articles, of which 597 articles met inclusion criteria. A total of 58 social functioning measures were identified. With some exceptions, reports on reliability and validity were scarce, and only one measure integrated social media social activity.

**Conclusions:**

The ecological validity of social functioning measures is challenged by the lack of assessment of social media activity, as it fails to reflect an important aspect of the current social reality of persons with psychosis. Measures should be revised to include social media activity and thus avoid the clinical consequences of inadequate assessment of social functioning.

**Trial Registration:**

International Prospective Register of Systematic Reviews (PROSPERO) CRD42017058514; http://www.crd.york.ac.uk/PROSPERO/display_record.php?ID=CRD42017058514

## Introduction

Social functioning impairment is a core dimension of psychotic disorders [1]. Thus, measures of social functioning are crucial for clinical assessment, prognosis, and outcome. Research indicates high engagement with social media platforms and associated social interaction in subjects with psychosis and those at ultrahigh risk (UHR) for psychosis states, including friendship formation and overcoming barriers associated with having severe psychiatric symptoms [2-5]. Social media activity should therefore be included as part of social functioning measures.

In an Australian national survey, more than one-third of adults with psychosis rated social functioning issues as their greatest challenge for the future [6]. Long-term deficits in social functioning have been linked to negative symptoms, such as social withdrawal, apathy, and avolition [7,8], as well as impaired social cognitive capacities, including capacity for mentalization and theory of mind [5,9]. Similar findings have been found for UHR populations; when compared to healthy controls, they show both higher levels of baseline social decline and lower levels of quality of life [10-14]. Conversely, good social functioning has been identified as a robust predictor of recovery [15-18].

Empirical research on social functioning largely originates from standardized questionnaires based on two dimensions [19]. The objective dimension encompasses the ability to meet social roles, such as employability and being a spouse, a family member, or a friend, combined with socioeconomic measures, such as finances and housing [20]. These measures are easily quantifiable and can thus be replicated [20]. The subjective dimension comprises self-reported measures of social roles and measures of satisfaction with family life, recreational activities, and life as a whole [20]. Ratings on both objective and subjective measures are found to correlate with prognosis, course development, and outcome [21].

Since the advent of Facebook in 2004, social media is exponentially more often involved in establishing and maintaining social networks [22-24]. Globally, there are approximately 2.6 billion registered social media profiles and the number is expected to grow by an additional 400 million over the next three years [25]. In 2015, in the United States, more than 75% of people used social media compared to 7% a decade ago, and 92% of adolescents went online daily [26]. Nonetheless, the conceptualization of social media participation as a dimension of social functioning is underdeveloped. At face value, when compared with offline contact, social media platforms represent radically evolving platform structures and a more asynchronistic kind of communication. These are technology-mediated tools that enable individuals to share, exchange, and create ideas, images, and information through online communities and networks [27-29].

Despite having fewer or less-frequent social contacts outside social media, individuals with psychosis or those at UHR for psychosis are heavy consumers of social media when compared to peers of the same age [30-34]. The Internet has become an influential source of mental health information for people with psychosis [28] and, thus, social media and digital devices have been utilized to support mental health care [32-35] and destigmatization campaigns [36]. Particularly for the youngest age group with psychosis and those at UHR for psychosis, there has already been social media-based interventions developed that are targeted on psychological, functional, and social recovery [37,38].

*Science and technology studies* aim at offering a comprehensive understanding of the interaction between science, technology, and society [39]. According to this framework, technologies may fundamentally alter societal dynamics, influencing communication. Moreover, post-normal science (PNS) is a perspective emphasizing the value of direct stakeholder involvement in practices where facts are uncertain and stakes are high [40], as they arguably are in psychosis. If measures of social functioning in psychosis do not embody the fundamental changes caused by technological innovations and do not consult target groups directly, they run the risk of low ecological validity.

The main objective of this study was to examine whether measures of social functioning in psychosis and UHR for psychosis include the assessment of social behavior on social media. It also compared the validity and reliability of reported measures of social functioning.

## Methods

This review followed the Preferred Reporting Items for Systematic Reviews and Meta-Analyses (PRISMA) guidelines [41] to ensure comprehensive and transparent reporting of methods and results. The protocol was registered at the International Prospective Register of Systematic Reviews (PROSPERO) in March 2017 (registration number: CRD42017058514).

### Search Strategy

Two independent authors (JB and WTVH) conducted a search using the following electronic databases: Epistemonikos, Cochrane Database of Systematic Reviews, Database of Abstracts of Reviews of Effects (DARE), MEDLINE, Embase, and PsycINFO. The search terms used were as follows: (“psychosis” or “psychoses” or “psychotic*” or “schizo*”) AND (“social*” or “psychosocial*” or “communit*” or “peer*” or “famil*” or “friend*”). Specific search terms were added to capture social media activity (eg, the Medical Subject Headings [MeSH] term “social media”; see Multimedia Appendix 1 for model search). The search queries were reviewed by an information scientist and were limited to title, abstract, keywords, and subject headings. In addition, a manual literature search was performed using reference lists of reviews and meta-analyses. In cases of doubt, the full-text paper was read to determine eligibility. Papers published between January 2004 and February 2019 were included. The last search was conducted on February 15, 2019.

### Eligibility Criteria

The included articles were required to meet all of the following criteria:

1. Empirical study published in the English language in a peer-reviewed journal.
2. The study included a measure of objective or subjective offline (ie, non-Web-mediated contact) and/or online social functioning (ie, Web-mediated contact).
3. The social functioning measure had to be used in samples meeting criteria (ie, Diagnostic and Statistical Manual of Mental Disorders [DSM] or International Classification of Diseases [ICD]) for a psychotic disorder or UHR for psychosis.
4. The study was published between January 2004 and February 2019. Facebook was launched as the first large-scale social media platform in 2004 and, therefore, it is highly improbable that studies conducted prior to 2004 would have included measures of social media activity.

### Exclusion Criteria

Articles were excluded if the only functioning assessed by the measure was one of the following:

1. Premorbid functioning measures.
2. Global functioning measure.
3. Performance-based skills assessment.
4. Studies exclusively dealing with social relationships, including social functioning, between study participants and professionals.

### Data Collection

All potential studies were exported into a reference citation manager, EndNote (Clarivate Analytics), before removing duplicates. Two independent reviewers (JB and WTVH) separately performed the screening of titles and abstracts, full-text analysis, and selection of social functioning measures. Disagreements were resolved through discussion until consensus was reached. A third reviewer (SP) was available to resolve disagreements. Finally, the list of included and excluded measures was sent to an independent auditor (HJS) for critical evaluation. The kappa coefficient was used to assess the level of agreement of the two independent reviewers for the selection of included and excluded measures.

### Analytic Methods and Data Extraction Procedure

A narrative descriptive synthesis was performed for the included articles. The data extraction procedure was performed in two steps. First, subjective and objective measures of social functioning across different social domains (ie, work, community functioning, socioeconomic status, etc) for both offline and online engagements were identified. Second, the content, quality, and psychometric properties, with a particular focus on whether measures assessed social media activities and interactions, were examined and assessed, including validity and reliability statistics of the measures. Since the selection of screened and included articles was extensive, validation literature was sourced directly from the reviewed articles. In addition, a manual search was performed for each individual measure to identify further validation literature.

## Results

### Search Results

**Figure 1:**
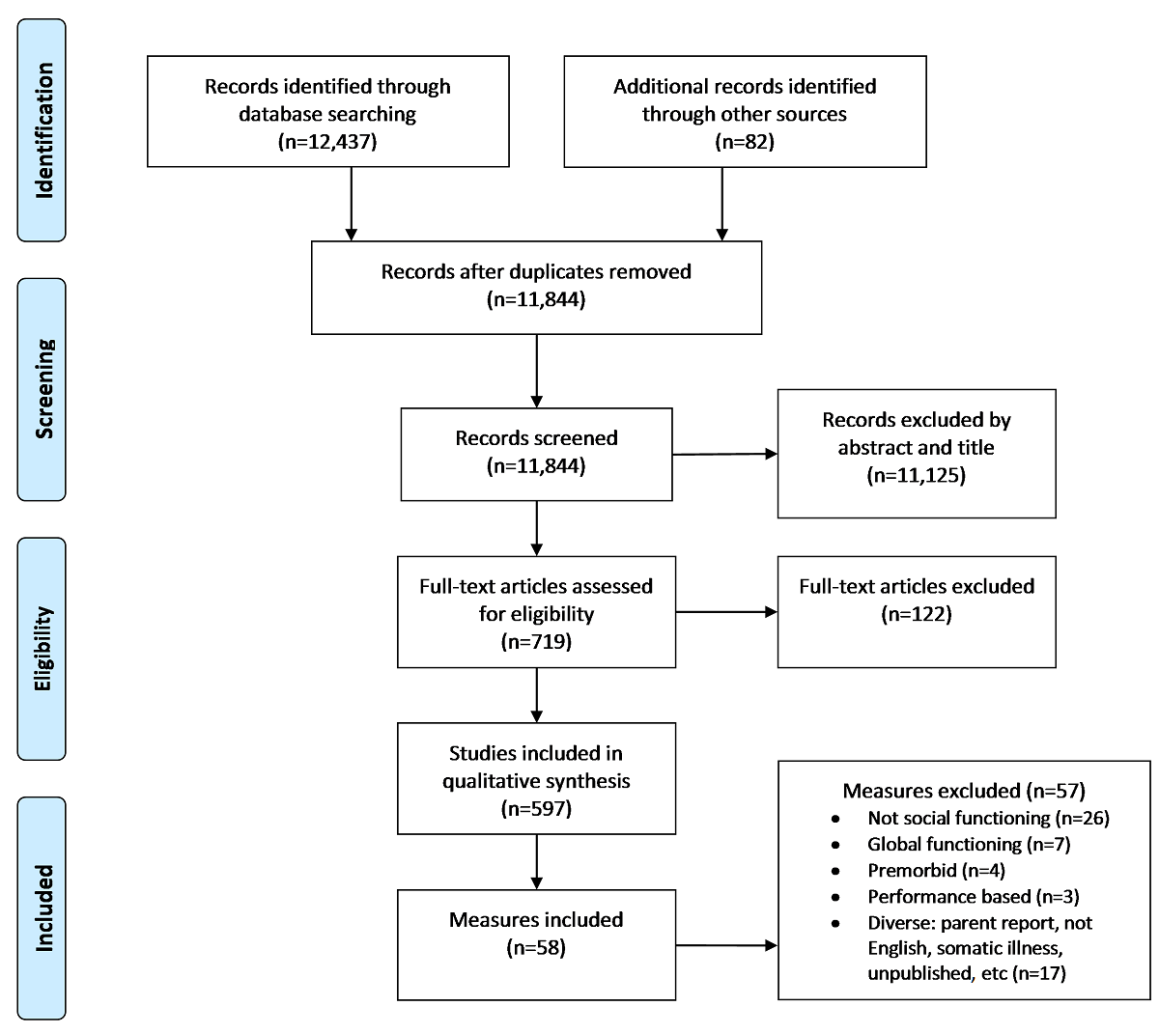
Flow diagram of the reviewing process according to the Preferred Reporting Items for Systematic Reviews and Meta-Analyses (PRISMA) guidelines.

The electronic search returned 12,437 articles. After duplicates were removed, there were 11,844 articles, of which 671 were reviews or meta-analyses: 178 articles from the Cochrane Database of Systematic Reviews and 493 from Epistemonikos. A hand search of reference lists of reviews and meta-analyses returned a further 82 articles. Full-text evaluation was conducted for 719 articles, of which 597 met the inclusion criteria and were included for the final synthesis. From the 597 articles, 58 measures were identified: 41 (71%) social functioning and 17 (29%) quality-of-life measures that included assessment of social functioning. Interrater reliability (ie, agreement between independent reviewers) for inclusion of measures was high (k=.83). Details of the search results are summarized in Figure 1.

### Frequently Used Social Functioning Measures

Details of the included 58 measures of social functioning are summarized in Tables 1 and 2. The three most frequently used measures were the Social Functioning Scale (78 references), the Quality-of-Life Scale (67 references), and the World Health Organization Quality of Life Brief Version (WHOQOL-BREF) (57 references). Several measures (eg, the Social Adjustment Scale II and the Global Functioning-Social Scale) had been used to address social functioning in UHR populations. Although developed for young people, none of these measures were exclusively used in UHR populations.

**Table 1:** Included social functioning measures (N=41).

Measure	Author, year, number of scale citations^a^	Description	Validation samples	Validity	Reliability^b^	Social media (Yes or No)
Assessment of Communication and Interaction Skills	Forsyth et al, 1999, 1	Observer-rated 20 items, 4-point Likert response format Three domains: physicality, information exchange, and relations Gathers data on skill as it is exhibited during performance in an occupational form and/or within a social group context	Psychosis and general psychiatry	Good internal and construct validity [43,44] Good discriminant validity [43,44]	Good interrater reliability [43,44]	No
Behavior and Symptom Identification Scale	Eisen et al, 1999, 1	Self-report 32 items, 4-point Likert response format Five domains: relationship (self and others), daily living and role performance, depression and anxiety, impulsive and addictive behavior, and psychosis	Psychosis, inpatients and outpatients	Questionable internal and construct validity; poor for psychosis [45] Good discriminant validity; unacceptable for psychosis [45] Good sensitivity to change [45]	Good interrater reliability [45]	No
Client’s Assessment of Strengths, Interests and Goals	Wallace et al, 2001, 2	Self-report and informant-report 102 items, 5-point Likert response format Six domains: current social and independent living skills, medication compliance and side effects, quality of life, quality of treatment, symptoms, and performance of unacceptable community behaviors	Psychosis and general psychiatry	Questionable concurrent validity Good discriminant validity [46]	Good test-retest reliability Questionable interrater reliability Good internal consistency [46]	No
Community Adjustment Form	Stein & Test, 1980, 7	Observer-rated semistructured interview 140 items, 10-item scale 17 domains (eg, living situation, work and social functioning, family involvement, and medication use) Also includes an observer-rated, 10-item scale of prosocial behaviors	Psychosis	No data available	Excellent interrater reliability [47]	No
Disability Assessment Schedule—II	WHO^c^, 2010, 31	Observer-rated 12, 36, or 97 items; scoring based on all available information (eg, patient´s written records or data from informants) Six domains: understanding and communicating, getting around, self-care, getting along with others, household and work activities, and participation in society Several versions: DAS^d^, DAS-II-sv^e^, SDSS^f^, and WHO-DAS^g^	General psychiatry	Moderate concurrent validity [48]	Good internal consistency [48] Excellent test-retest reliability [48]	No
First Episode Social Functioning Scale	Lecomte et al, 2014, 5	Self-report 34 items, 4-point Likert response format Eight domains: independent living skills, interacting with people in different contexts, social activities, intimacy, friendships, family relations, work, and school Perceived ability and actual behavior rated for each item	Psychosis	Good convergent validity [42] Good discriminant validity [42]	Good sensitivity to change [42]	Yes
Functional Assessment Short Test	Rosa et al, 2007, 1	Self-report 24 items, 4-point Likert response format Six domains: autonomy, occupational functioning, cognitive functioning, financial issues, interpersonal relationships, and leisure time	Bipolar disorder	Good concurrent validity Questionable discriminant validity [49]	Good test-retest reliability [49] High internal consistency [49]	No
Functional Remission of General Schizophrenia	Llorca et al, 2009, 5	Observer-rated 19 items, 5-point Likert response format Six domains: daily life, activities, relationships, quality of adaptation, health, and treatment	Schizophrenia	Good concurrent validity [50]	Good internal consistency [50]	No
Global Assessment of Relational Functioning	Dausch BM et al, 1996, 1	Observer-rated 20 domains of relationship functioning, 100-point scale (81-100 indicates satisfying functioning)	Bipolar disorder	Good concurrent validity Good discriminant validity [51]	Good-to-high interrater reliability [51] Good internal consistency [51]	No
Global Functioning—Social	Cornblatt B et al, 2007, 22 (5 UHR^h^)	Observer-rated Seven probe questions, 10-point Likert response format Assesses levels of social contact and friendships outside of the family	Psychosis	Good construct validity [52]	Good interrater reliability [52]	No
Groningen Social Disability Schedule	Wiersma, 1988, 8	Observer-rated semistructured interview 5-point Likert response format Eight domains: self-care, relationship with the family, relationship with a partner, friendship, parental role, citizenship, leisure activities, and work and occupation	General psychiatry	Poor-to-excellent sensitivity to change [53] Good discriminant validity [54]	Good interrater reliability Questionable test-retest reliability [53]	No
Health of the Nation Outcome Scale	Wing et al, 1998, 8	Observer-rated 12 items, 4-point Likert response format Aggression, self-harm, drug and alcohol use, cognitive problems, physical illness and disability, hallucinations and delusions, depression, other symptoms, social relationships, activities of daily living, residential environment, and day-time activity	General psychiatry	Good concurrent validity [55] Good discriminant validity [55]	Poor-to-good interrater reliability [55] Poor-to-acceptable test-retest reliability [55]	No
Index of Social Competence	McConkey R & Walsh J, 1982, 1	Observer-rated 15 items of ability Six domains: community skills, self-care skills, communication skills, time, money, and additional handicaps	General psychiatry	No data available	Good interrater reliability [56]	No
Inventory of Interpersonal Problems	Horowitz et al, 1988, 1	Self-report 32 items, 4-point Likert response format Nine domains: domineering or controlling, vindictive or self-centered, sociable, intimate, submissive, responsible, nonassertive, self-sacrificing, and intrusive	Community: nonclinical	No data available	Acceptable-to-good test-retest reliability [57] Good internal consistency [57]	No
Interview Schedule for Social Interaction	Henderson S et al, 1980, 2	Observer-rated 53 items, individual summary scores for each domain Four domains: availability of close relationships, adequacy of above, availability of friendships, and adequacy of above	General population	Good face validity [58]	Acceptable test-retest reliability [58] Acceptable internal consistency [58]	No
Life Skills Profile	Parker et al, 1991, 14	Observer-rated 39 items, 4-point Likert response format Five domains: ability for self-care, turbulent behavior (reverse scored), sociability, communication, and responsibility	Psychosis, inpatients and outpatients	Questionable construct validity [59]	Good interrater reliability [59] Good test-retest reliability [59]	No
Multnomah Community Ability Scale	Barker et al, 1994, 12	Observer-rated 17 items, 5-point Likert response format Four domains: assessment of interference with functioning, adjustment to living, social competence, and behavioral problems	General psychiatry, chronic patients	No data available	Good interrater reliability [60] Good test-retest reliability [60]	No
Personal and Social Performance	Morosini et al, 2000, 55	Observer-rated Single-item, 100-point response format (score determined by domain score range) Four domains (6-point response format per domain): socially useful activities, personal and social relationships, self-care, disturbing and aggressive behaviors	Psychosis	Good construct validity [61]	Good interrater reliability [61] Excellent test-retest reliability [61]	No
Provision of Social Relations Scale	Turner et al, 1983, 2	Self-report 15 items that measure social relationships with family and friends, 5-point Likert response format	Schizophrenia, bipolar disorder, and healthy controls	No data available	Good test-retest reliability [62]	No
Psychosocial Functioning Scale	Valencia et al, 1989, 1	Self-report 35 items, 5-point Likert response format Five domains: occupational, social, money management, marital, and familial	General psychiatry	Good construct validity	Good internal consistency [63]	No
Recovery Assessment Scale	Corrigan et al, 1999, 2	Self-report 41 items, 5-point agreement scale Four domains: doing things I value, looking forward, mastering my illness, and connecting and belonging	General psychiatry, chronic patients	Good concurrent validity [64]	Good test-retest reliability [64]	No
Rehabilitation Evaluation Hall and Baker	Baker R & Hall JN, 1988, 1	Observer-rated 22 items, 9-point Likert response format Two domains: deviant behavior and general behavior General Behavior subscale (only subscale relevant for the cited study): 15 items Five domains: social activity, speech disturbance, speech skills, self-care skills, and community skills	General psychiatry	Good criterion validity [65] Good discriminant validity [65]	Good interrater reliability [65]	No
Role Functioning Scale	Goodman et al, 1993, 7	Observer-rated Four items, 7-point Likert response format Four domains: work, independent living and self-care, immediate social network relationships, and extended social network relationships	Psychosis and depression	Good discriminant validity [66]	Poor-to-good interrater reliability [66] Good test-retest reliability [66] Good internal consistency [66]	No
Schizophrenia Social Functioning Index	Padmavathi R, 1995, 2	Observer-rated 17 items, 5-point Likert response format Four domains: self-concern, occupational role, role in family, and other social roles Each section has several subsections covering different areas of social functioning	Schizophrenia and relatives	Good concurrent validity [67]	Good interrater reliability [67] Good test-retest reliability [67] Acceptable internal consistency [67]	No
Self Evaluation and Social Support—Schizophrenia version	Humphreys et al, 2001, 1	Observer-rated Five domains: social and recreational, occupational, relationships, parenting, and homemaking Questions in these sections involve both perceived competence and commitment in each possible role, and responses are used in an overall rating; also, sections covering self-evaluation of personal attributes and self-acceptance	Psychosis	No data available	Acceptable-to-good interrater reliability [68] Poor test-retest reliability [68]	No
Short Form Health Survey—36	Ware & Donald, 1992, 12	Observer-rated or self-report 36 items, 100-point response format Eight domains: physical functioning, role limitations due to physical health problems, bodily pain, general health perceptions, vitality, social functioning, role limitation due to emotional problems, and general mental health	Psychosis	Moderate discriminant validity [69]	Good test-retest reliability [69]	No
Social Adaptation Self-Evaluation Scale	Bosc M et al, 1997, 4	Self-report 21 items, 4-point Likert response format Four domains: social, familial, occupational, and environmental functioning	Major depression	Acceptable sensitivity to change [70]	Poor test-retest reliability [70] Good internal consistency [70,71]	No
Social Adaptive Functioning Evaluation	Harvey et al, 1997, 1	Observer-rated 17 items, 5-point Likert response format Three domains: impulse control, instrumental and self-care, and social functions	Schizophrenia	Good convergent validity [72] Good discriminant validity [72]	Good interrater reliability [72] Good test-retest reliability [72]	No
Social Adjustment Inventory for Children and Adolescents	Gammon et al, 1987, 1 (UHR)	Observer-rated interview Multiple domains: functioning in school, spare time activities, and interactions with peers and family	Children and adolescents of parents with and without major depression	Questionable-to-poor convergent validity [73]	Poor interrater reliability [71,73] Good-to-poor internal consistency [73]	No
Social Adjustment Scale II	Paykel et al, 1971, 27 (1 UHR)	Observer-rated semistructured interview 52 items, 5-point Likert response format Eight domains: work, domestic relationship, parental role, relationship with external family, social and leisure activities, sexual activity, romantic involvement, and personal well-being Self-report version [74]	Depression	Poor-to-acceptable convergent validity [74]	No data available	No
Social Behaviour Scale	Wykes & Sturt, 1986, 16	Observer-rated 21 items, 5-point Likert response format Six domains: occupation, behavioral problems, personal self-care, leisure activities, performance and expectations, and communication skills	General psychiatry	Good discriminant validity [75] Good concurrent validity [75]	Good interrater reliability [76] Good interinformant reliability [76] Good test-retest reliability [76]	No
Social Functioning Questionnaire	Tyrer et al, 2004, 1	Self-report Eight-item assessment of perceived social functioning, score 0-24 Developed from the Social Functioning Scale	Psychiatric outpatient (nonpsychotic)	No data available	Good interrater reliability [77] Good test-retest reliability [77] Good internal consistency [77]	No
Social Functioning Scale	Birchwood et al, 1990, 78	Self-report or observer-report 79 items, 4-point Likert response format Seven domains: social engagement, interpersonal behavior, prosocial activities, recreation, independence-competence, independence-performance, and employment and occupation	Psychosis	Good construct validity [78] Good discriminant validity [78] Good convergent validity [78]	Good interrater reliability [78]	No
Social Integration Survey	Kawata AK & Revicki DA, 2008, 1	Self-report or informant-report 62 items, 4-6-point Likert response format Five domains: social perception, work interactions, social skills, social cognition, and daily living skills or self-care	Schizophrenia	Good discriminant validity [79]	Good internal consistency [79] Poor interrater reliability [79]	No
Social and Occupational Functioning Assessment Scale	American Psychiatric Association, 1994, 35	Observer- or self-report Single-item, 100-point response form	General psychiatry	Acceptable sensitivity to change [80]	No data available	No
Social Occupational Functioning Scale	Saraswat N, 2006, 2	Observer-rated 15 items, 5-point Likert response format Three domains: adaptive living skills, social appropriateness, and interpersonal skills	Schizophrenia	Acceptable concurrent validity [81] Acceptable criterion validity (positive and negative symptom total score) [81] Acceptable discriminant validity [81]	High internal consistency [81] Good test-retest [81]	No
Social Role Functioning Test	McPheeters H, 1984, 1	Observer-rated 28 items, 7-point Likert response format Three domains: work productivity, independent living, and social network relationships (immediate and extended)	General psychiatry	No data available	Good internal consistency [82]	No
Specific Levels of Functioning	Schneider & Struening, 1983, 19	Observer-rated 43 items, 5-point Likert response format Six dimensions: physical functioning, personal care skills, interpersonal relationships, social acceptability, activities, and work skills	Psychosis and general psychiatry	No data available	Excellent internal consistency [83] Excellent interrater reliability [83]	No
Strauss Carpenter Level of Functioning	Strauss & Carpenter, 1977, 10	Observer-rated Four items, 5-point Likert response format Four domains: symptomatology, work, social contacts, and function	Not stated	No data available	No data available	No
Strauss Carpenter Outcome Scale	Strauss & Carpenter, 1972, 7	Observer-rated Four items, 49-point Likert response format Four domains: social activities, work, independent living, and hospitalization	Schizophrenia	No data available	No data available	No
Time Budget Measure	Jolley et al, 2006, 1	Observer-rated Diary-based measure (28 time blocks for the week), score range 0-112	Psychosis	Acceptable convergent validity [84]	Good interrater reliability [85] Good test-retest reliability [85]	No

^a^Number of citations the scale has gotten throughout the years, which indicates the scale popularity and impact.

^b^Reliability: 1 (perfect reliability), ≥.9 (excellent reliability), ≥.8<.9 (good reliability), ≥.7<.8 (acceptable reliability), ≥.6<.7 (questionable reliability), ≥.5<.6 (poor reliability), <.5 (unacceptable reliability), 0 (no reliability).

^c^WHO: World Health Organization.

^d^DAS: Disability Assessment Schedule.

^e^DAS-II-sv: Disability Assessment Schedule—II: Schizophrenia Version

^f^SDSS: Social Disability Screening Schedule.

^g^WHO-DAS: World Health Organization Disability Assessment Schedule

^h^UHR: ultrahigh risk.

**Table 2:** Included quality-of-life measures (N=17).

Measure	Author, year, number of scale citations^a^	Description	Validation samples	Validity	Reliability^b^	Social media (Yes or No)
Assessment of Quality of Life	Hawthorne et al, 1999, 2	Self-report 47 items, 4-point Likert response format Five domains: illness, independent living, social relationships, physical senses, and psychological well-being	Clinical and community sample	Good discriminant validity [86]	Good internal consistency [86]	No
Health Related Quality of Life	Nelson et al, 1987, 3	Self-report 5-point Likert response format Nine global questions, each illustrated with drawings to measure the following domains: physical fitness, pain, feelings and emotions, daily activities, social activities, change in health, overall health, social support, and overall quality of life.	Mainly chronic illnesses	Good face validity [87]	Good test-retest reliability [88] Good interrater reliability [88]	No
Lancashire Quality of Life Profile	Oliver, 1991, 3	Observer-rated 100 items, 7-point Likert response format 10 domains: living situation, leisure and social participation, health, finances, family relations, safety, positive esteem, negative esteem, framework, and fulfilment	General psychiatry	Moderate-to-good concurrent validity [89]	Moderate-to-good internal consistency [89] Good test-retest reliability [89]	No
Manchester Short Assessment of Quality of Life	Priebe et al, 1989, 23	Observer-rated clinical interview 25 items, 7-point Likert response format 12 domains with three subscales: these include stable personal patient details, personal details that may change over time (eg, education), and questions that must be asked at each assessment, including both objective and subjective items concerning quality of life and social life	Psychosis and students	Good concurrent validity [90]	Good internal consistency [91]	No
Modular System for Quality of Life	Pukrop R et al, 2000, 3	Self-report 47 items, 7-point Likert response format Seven domains: physical health; vitality; and psychosocial, affective, material, spare time, and general quality of life	Schizophrenia and general population	Poor discriminant validity [92]	Good internal consistency [92] Good test-retest reliability [92]	No
Sickness Impact Profile	Pollard et al, 1976, 1	Self-report 48 items (short version), individual category scores Four domains: sleep and rest, home management, contact with family and friends, and leisure activities	Psychiatric samples and somatic samples	High internal consistency [93] Good concurrent validity [94]	High test-retest reliability [93] High interrater reliability [93]	No
Quality of Life Enjoyment and Satisfaction Questionnaire—Short Form	Endicott et al, 1993, 8	Observer-rated 59 items, 5-point Likert response format Five domains: general activities, physical activities, emotional functioning, recreational activities, and social relationships	Psychosis and depression	Poor-to-moderate discriminant validity [95]	Good internal consistency [96] Moderate test-retest reliability [96,97]	No
Quality of Life Index	Ferrans CE & Powers MJ, 1985, 1	Self-report 32 items, 6-point Likert response format Four domains: health and functioning, social and economic, psychological and spiritual, and family	Schizophrenia and healthy controls	Good convergent validity [98]	Excellent internal consistency [98]	No
Quality of Life Interview	Lehman, 1983, 29	Observer-rated semistructured interview 143 items (brief versions: 33 or 78 items) Eight domains: accommodation, family, social relations, leisure, safety, finances, physical health, and mental health	Psychosis and general psychiatry	Good construct validity [99]	Good-to-acceptable internal consistency [99] Poor-to-good test-retest reliability [99]	No
Quality of Life Inventory—74	Frisch MB, 1992, 2	Self-report 17 items, 4-point Likert response format	General psychiatry, undergraduates, and forensic	Good convergent validity [100] Good construct validity [100]	Good test-retest reliability [100] Good internal consistency [100]	No
Quality of Life Scale	Heinrichs et al, 1984, 67	Observer-rated semistructured interview 21 items, 7-point Likert response format Four domains: intrapsychic foundations, interpersonal relations, instrumental role, and common objects and activities	Psychosis and general psychiatry	Poor-to-good convergent validity [101]	Excellent interrater reliability [101]	No
Quality of Well-Being	Kaplan et al, 1978, 2	Observer-rated Preference weight for each domain Three domains: mobility, physical activity, and social activity	Chronic somatic illness	Good discriminant validity [102,103] Good convergent validity [102,103]	No data available	No
Satisfaction With Life Scale	Test et al, 2005, 6	Self-report 18 items, 5-point Likert response format Four domains: living situation, work, social relationships, and self and present life	Schizophrenia and general psychiatry	Good construct validity [104] Good concurrent validity [104]	Good internal consistency [104]	No
Schizophrenia Quality of Life—18	Boyer et al, 2010, 1	Self-report 41 items, index scores from 0 to 100 Eight domains: psychological well-being, self-esteem, family relationships, relationships with friends, resilience, physical well-being, autonomy, and sentimental life	Schizophrenia	Good-to-unacceptable concurrent validity; great scale variability [105]	Good-to-acceptable internal consistency [105] Good-to-acceptable test-retest reliability [105]	No
Schizophrenia Quality of Life Scale	Wilkinson et al, 2000, 10	Self-report 30 items, 5-point Likert response format Three domains: psychosocial, motivation and energy, and symptoms and side effects	Schizophrenia	Good construct validity [106]	Good internal consistency [106] Good test-retest [106]	No
Wisconsin Quality of Life Index	Becker, 1993, 4	Self-report 113 items, 5-point Likert response format Nine domains: general life satisfaction, activities and occupations, psychological well-being, physical health, social relations and support, economics, activities of daily living, symptoms, and goal attainment	Schizophrenia and general psychiatry	Good convergent validity [107]	Good-to-acceptable internal consistency [107] Good test-retest reliability [107]	No
WHOQOL-BREF^c^	WHO Quality of Life Group, 1998, 57	Self-report 268 items, 5-point Likert response format Four domains: physical, psychological, social, and environmental	Psychosis and general psychiatry	Poor-to-moderate construct validity [108]	Good internal consistency [108]	No

^a^Number of citations the scale has gotten throughout the years, which indicates the scale popularity and impact.

^b^Reliability: 1 (perfect reliability), ≥.9 (excellent reliability), ≥.8<.9 (good reliability), ≥.7<.8 (acceptable reliability), ≥.6<.7 (questionable reliability), ≥.5<.6 (poor reliability), <.5 (unacceptable reliability), 0 (no reliability).

^c^WHOQOL-BREF: World Health Organization Quality of Life Brief Version.

### Structure and Administration of Measures

A total of 35 out of 58 measures (60%) were primarily observer-rated, while 23 (40%) were primarily self-reported. The completion time ranged from 10 minutes (ie, Social Functioning Questionnaire) to 60 minutes (ie, Social Adjustment Scale). Most of the social functioning and quality-of-life measures used a Likert response format (40/58, 69%). Most measures assessed behaviors, not perceived ability, related to physical forms of social functioning, such as face-to-face or telephone contact with friends and family. There was great variability in how comprehensive measures reported on social functioning characteristics, ranging from the First Episode Social Functioning Scale (FESFS) with nine subscales to those who reported a few items (eg, part of a single subscale) of social functioning. Also, quality-of-life measures typically concentrated more on subjective evaluations of general life domains and were thus less focused on social functioning. The FESFS was the only measure to include an assessment of social activity on social medial; this is evaluated in a separate section below.

### Psychometric Properties of the Measures

Out of all 58 included measures; 32 (55%) had previously been validated in patients with psychosis, 16 (28%) in a general psychiatric or clinical and community sample, 2 (3%) in a sample of patients with bipolar disorder, 2 (3%) in a sample of patients with depression, 2 (3%) in a sample of patients with somatic illness, 2 (3%) in a nonclinical sample, 1 (2%) in a sample of adolescents of parents with and without major depression, and 1 (2%) did not record any sample information. More data were available for reliability (53/58, 91%) than for validity (47/58, 81%). In general, lack of information prevented a comprehensive evaluation of the psychometric properties of most measure instruments. Theoretical foundation and construct validity was particularly poorly reported. When psychometric properties were reported, measures showed overall good validity and reliability regarding offline social functioning. The Social Functioning Scale, the Groningen Social Disability Schedule, and the Health of the Nation Outcomes Scale are examples of measure instruments with comprehensive reporting of this type of social functioning.

### Measure Assessing Social Activity on Social Media

The FESFS was developed in 2014 [42] by the authors listing activities based on their experience with people with early psychosis and on reviews of existing measures of social functioning [40]. The FESFS is designed to measure social functioning in young people in the early stages of psychosis and was the only measure instrument identified in this review as addressing social activity on social media. The scale can be administered as observer-rated or self-report, with each item rating behavior—focus on frequency—and perceived individual ability. The FESFS comprises 34 items distributed on nine subscales assessing various domains of social functioning. The item language was intentionally constructed to fit the target group (eg, “hanging out with buddies” and “chatting on the net”). Two items, respectively from the items *Friends and activities* and *Living skills*, address social media activity explicitly: “I am really good in solo activities such as going to the gym, going to the movies, chatting on the net, taking lessons (music, painting, etc)” and “I am comfortable using the phone, Internet, or email to communicate.” The scale is cited five times, of which three of the cited articles include the measure developers as authors.

Scale validation was based on the self-report version. The validation sample included 203 people, with an average age of 24.5 years, diagnosed with a schizophrenia spectrum psychotic disorder, and with an average of 12.7 years of education. The nine factors showed good internal consistency, ranging from .63 to .80. Good convergent and discriminant validity, as well as good sensitivity to change, were also demonstrated. Three subscales had an inverse correlation with negative symptoms.

## Discussion

### Measures Should Include Contemporary Social Reality

Due to technological innovation and rapid alterations in the norms of social media usage, any instrument designed to measure social functioning, including social media activity, should encapsulate contemporary trends. The main finding of this review was that current measures of social functioning almost exclusively comprise offline social activity, with the sole exception of the FESFS, as discussed in a separate section below. This limitation is likely to reflect the time of development of currently used measures, as most were developed before the launch of the Internet in 1992, and only eight measures were developed or revised after the advent of Facebook in 2006. Many of these scales have good psychometric properties, which may be a good starting point if they were revised to include measures of social media activity. It is notable that the first measures developed were based on chronic inpatients (eg, the Interview Schedule for Social Interaction). However, there is now an emphasis on early intervention to target quality of life and younger early-stage patients, as opposed to chronic inpatients [109], and current measures fail to capture an important aspect of the current social context.

It is worth discussing whether the two most widely used categories of measures—social functioning and quality of life—are expedient. For instance, a number of the measures within these categories address social participation, while others address the more narrowly defined concept of social skills. In practice, then, choosing a measure from either category for evaluative purposes could potentially influence interpretations of findings.

Further, regarding validity, while the original psychometric assessment of some measures show good reliability and validity, they may lack ecological validity. For example, leaving out assessment of social media activity may lead to low scores on social functioning among young people with psychosis and, thus, increase the likelihood of false positives. Moreover, the core negative symptom of social withdrawal [8] may manifest differently in a social media context compared to an offline context. There is also a risk of social media addiction, negative social comparison, cyberbullying, as well as it being used to exclude real-life contacts [27,110], with potential negative consequences on illness course, outcome, and quality of life. Online social functioning measures should aim to be sensitive to these types of matters. Also, they should track symptom levels [2], change in social participation, and support that unfolds online [3]. In this regard, a survey found that adults with schizophrenia were as likely as adults without mental illness to form social relationships online, despite having fewer offline relationships, lower income, and less Internet access [4]. Compensating for symptoms that people with psychosis themselves experience that interfere with socializing in face-to-face encounters [111] may be a fruitful remedy for some of the obstacles associated with the enhanced levels of toxic loneliness and stigma associated with psychosis populations [5]. This type of information would also be important for treatment timing and tailoring.

### Social Media Assessment

The FESFS represents an attempt to address contemporary forms of social functioning, including online activity. Additionally, the scale assesses both behavior and ability, which make a more nuanced assessment possible. However, the scale has fundamental limitations. The validation sample has an average age of 24.5 years, which is relatively high when aiming at early psychosis and UHR of psychosis. The subscales related to work and education are not satisfactorily validated, as only a small part of the validation sample was working or studying. Test-retest reliability for the scale has not been conducted and neither factorial structure nor construct validity has been confirmed. In addition, only the self-report version has been validated. Furthermore, the scale has only been cross-validated across context to a very limited extent [112]—as opposed to, for example, the Personal and Social Performance or the Psychosocial Functioning Scale—which implies uncertainty regarding robustness and usability. Further, the authors do not articulate a theoretical foundation for the scale, and scale content is derived from the scale authors’ own listing of experience-based domains of social functioning.

*Science and technology studies* is a highly influential theoretical framework analyzing the entanglements of science, technology, and society [39]. A basic premise in *science and technology studies* is that technological innovation affects society and human behavior in fundamental ways. Specific technologies, such as social media, do not merely add to the possibilities of communication, but changes the nature of communication processes. Consequently, attempts to include technology-mediated communication processes should start from the premise that these probably do not reflect nontechnological communication. Compared with face-to-face contact, social media represents radically evolving platform structures and a more asynchronistic form of communication. However, it is unclear whether social media platforms require extra social flexibility or if they are adaptable to facilitate communication for persons who may have limitations in face-to-face social skills, such as the limitations typically found for individuals with active psychosis. It has been suggested that individuals with mental health problems may use social media to seek support. When compared to face-to-face interaction, social media allows more time for reflection before acting [113].

The FESFS “chatting on the net” item is defined as a solo activity and yet this may not reflect the experience of social media by individuals. Social media includes virtual communities allowing users to create a public profile, interact with real-life and virtual friends, and make new acquaintances. Social media engagements often seem to be a fundamental social activity [114]. Also, the FESFS defines using the Internet or email communication as a living skill. However, it is difficult to equate these technological skills as being representative of social activity or functioning. While the FESFS has been the first measure to attempt to capture social media activity, the measure requires significant further development for validity of measurement of contemporary social media engagements.

### Future Research: Need for a Radical Change

The use of social media as a dimension of social functioning in psychosis is a complex issue and the knowledge base is limited. It is possible to explore social media behavior based on the most reliable and valid dimensions of currently available offline social functioning measures, such as the Social Functioning Scale. This scale provided the most comprehensive reporting of traditional psychometric properties for offline social functioning, including construct validity. With this scale, social skills or social behavior were registered as present or absent, thus removing the need for an evaluative decision. This could be a feasible starting point to track online social behavior. Some degree of social skill transfer between online and offline activities seems plausible. Additionally, it might be important to understand the relationship between more traditional measures of social skills and social media usage. In this regard, purely scientist-driven approaches have clear limitations. For example, the likely age gap between researchers and the target group of early psychosis, particularly the UHR segment, risks a lack of understanding of the social context. Therefore, a collaborative approach with the target group as codevelopers of the measure could remedy this shortcoming. The general omission of user involvement, which is highly prioritized and valued in most contemporary health care systems, is a major challenge to the validity of these measures [115]. We therefore propose a theoretical framework in which service users are involved, so as to explore social media as part of social functioning of young people with psychosis.

PNS was developed for interpreting and applying scientific results at the science-policy interface. PNS was tailored for situations where “facts [are] uncertain, values [are] in dispute, stakes [are] high, and decisions [are] urgent” [40]. The research field of social functioning in psychosis includes multiple theoretical perspectives, such as physiological, biological, evolutionary, social, and cultural perspectives. The complex nature of social functioning makes it difficult to indicate causality [1]. There are conflicts of interest causing tension between groups, such as the psychopharmaceutical industry, governments, professional associations, and user organizations [116-118]. The stakes are arguably high as social functioning impairment is regarded as a core symptom of serious mental illness, namely psychosis [119]. The PNS remedy is to communicate uncertainty, assess quality, and justify practice by including extended peer communities. In practical terms, the PNS framework ensures the inclusion of social components perceived as important by the target group. This will presumably lead to inclusion of new facets of social functioning that have been omitted by previous measures, and the risk of implementing outdated or ecologically invalid models is lowered.

Future reviews should take social media use or online activity into consideration when also evaluating social functioning measures in general patient populations. When developing and validating social functioning measures, researchers today should include social media activity: content, frequency, quality, and effects, both positive and negative.

### Strengths and Limitations

The strengths of the study are evident in the study protocol being publicly available (ie, PROSPERO) before conducting the review, thus ensuring transparency, and the review was conducted according to PRISMA guidelines [41]. In addition, the inclusion of studies was determined by two independent raters and showed high interrater reliability.

The main purpose of this review was to assess to what degree social functioning measures included assessments of any online social activity. Hence, we applied broad inclusion criteria to avoid ignoring any potential measures. A side effect of this strategy was the inclusion of some measures that were not tailored to specifically target social functioning in general or psychosis specifically.

The conclusions drawn in this review may have been influenced by several of the included studies not reporting relevant psychometric properties. Although only one of the identified instruments specifically assessed social media activity, it cannot be ruled out that respondents may answer generic questions about social functioning with social media activity in mind. Another limitation is that each individual study was not assessed for key sources of biases (eg, sample characteristics). However, in line with previous research [20], it seems warranted to conclude that some studies were based on small samples and that most instruments were constructed and tested within Anglo-American cultures. Grey literature was not included. This will typically raise the risk of reporting bias, implying that the included studies represent selective research dissemination [120]. However, it should be emphasized that the aim was to identify instruments with a high level of use within the field and that the search was conducted in several literature databases. The included studies did use samples with somewhat different characteristics (eg, sex, age, and level of symptomatology), which may violate the transitivity assumption and, thus, questions direct comparisons across included studies.

## Multimedia Appendix 1

Model search for replication.

## Abbreviations

DARE: Database of Abstracts of Reviews of Effects
DAS: Disability Assessment Schedule
DAS-II-sv: Disability Assessment Schedule—II: Schizophrenia Version
DSM: Diagnostic and Statistical Manual of Mental Disorders
FESFS: First Episode Social Functioning Scale
ICD: International Classification of Diseases
MeSH: Medical Subject Headings
PNS: post-normal science
PRISMA: Preferred Reporting Items for Systematic Reviews and Meta-Analyses
PROSPERO: International Prospective Register of Systematic Reviews
SDSS: Social Disability Screening Schedule
UHR: ultrahigh risk
WHO: World Health Organization
WHO-DAS: World Health Organization Disability Assessment Schedule
WHOQOL-BREF: World Health Organization Quality of Life Brief Version
