# Supplementary material for: Social Media and Social Functioning in Psychosis: A Systematic Review
Source: J Med Internet Res. 2019 Jun 28;21(6):e13957. doi: 10.2196/13957 (PMC6625220; doi:10.2196/13957)
Supplement: Supplementary file 1 [file jmir_v21i6e13957_app1.docx]

**Ovid Medline, Embase og PsycInfo**

Database: Ovid MEDLINE(R) and Epub Ahead of Print, In-Process & Other Non-Indexed Citations and Daily <1946 to February 15, 2019>

--------------------------------------------------------------------------------

1     exp Psychotic Disorders/ (49222)

2     exp Schizophrenia/ (99833)

3     (psychosis or psychoses or psychotic* or schizo*).ti,ab,kw. (176484)

4     or/1-3 (208362)

5     Social Media/ (5474)

6     (social* or psychosocial* or communit* or peer* or famil* or friend*).ti. (528527)

7     5 or 6 (531441)

8     4 and 7 (10612)

9     limit 8 to english language (9239)

10     limit 9 to yr="2004 -Current" (5140)

Database: Embase <1974 to 2019 February 15>, Ovid MEDLINE(R) and Epub Ahead of Print, In-Process & Other Non-Indexed Citations and Daily <1946 to February 15, 2019>, PsycINFO <1806 to February Week 1 2019> Search Strategy:

--------------------------------------------------------------------------------

1     exp Psychotic Disorders/ (308329)

2     exp Schizophrenia/ (353836)

3     (psychosis or psychoses or psychotic* or schizo*).ti,ab,kw. (566950)

4     or/1-3 (682710)

5     Social Media/ (25836)

6     (social* or psychosocial* or communit* or peer* or famil* or friend*).ti. (1497214)

7     5 or 6 (1512130)

8     4 and 7 (36097)

9     limit 8 to english language (31573)

10     9 use ppez (9239)

11     limit 10 to ep=20180101-20190218 [Limit not valid in Embase,PsycINFO; records were retained] (418)

12     limit 10 to ez=20180101-20190218 [Limit not valid in Embase,PsycINFO; records were retained] (465)

13     11 or 12 (492)

14     exp psychosis/ (418594)

15     (psychosis or psychoses or psychotic* or schizo*).ti,ab,kw. (566950)

16     14 or 15 (671101)

17     social media/ (25836)

18     (social* or psychosocial* or communit* or peer* or famil* or friend*).ti. (1497214)

19     17 or 18 (1512130)

20     16 and 19 (35462)

21     limit 20 to english language (31071)

22     21 use oemezd (12809)

23     limit 22 to dd=20180101-20190218 [Limit not valid in Ovid MEDLINE(R),Ovid MEDLINE(R) Daily Update,Ovid MEDLINE(R)

In-Process,Ovid MEDLINE(R) Publisher,PsycINFO; records were retained] (557)

24     exp PSYCHOSIS/ (418594)

25     (psychosis or psychoses or psychotic* or schizo*).ti,ab,id. (563090)

26     24 or 25 (668656)

27     social media/ or online social networks/ or online community/ (31944)

28     (social* or psychosocial* or communit* or peer* or famil* or friend*).ti. (1497214)

29     27 or 28 (1515362)

30     26 and 29 (35398)

31     limit 30 to english language (30988)

32     31 use psyh (9745)

33     limit 32 to up=20180101-20190218 (268)

34     13 or 23 or 33 (1317)

35     remove duplicates from 34 (1115)

36     35 not (23 or 33) (302)

37     35 not (13 or 33) (557)

38     35 not (13 or 23) (256)
